# Polyamine Homeostasis and Morphophysiological Responses to Salinity in *Dizygostemon riparius*: An Endemic Species from Brazilian ‘Cerrado’ Biome

**DOI:** 10.3390/biology14111494

**Published:** 2025-10-25

**Authors:** Jordanya Ferreira Pinheiro, Sérgio Heitor Sousa Felipe, Irislene Cutrim Albuquerque, Vitória Karla de Oliveira Silva-Moraes, Givago Lopes Alves, Marion Nayon Braga Soares, Juliane Maciel Henschel, Laíse Trugilio Moreira Marinho, Claudete Santa-Catarina, Diego Silva Batista, Fábio Afonso Mazzei Moura de Assis Figueiredo, Fabrício de Oliveira Reis, Tiago Massi Ferraz, Aldilene da Silva Lima, Thais Roseli Corrêa

**Affiliations:** 1 Programa de Pós-Graduação em Ciências Agrárias (PPGCIAG), Universidade Estadual do Maranhão (UEMA), São Luís 65055-310, MA, Brazil; jordanyaf.p@gmail.com (J.F.P.); albuquerqueiris0@gmail.com (I.C.A.); vitoriakarlaos@gmail.com (V.K.d.O.S.-M.); engivago@gmail.com (G.L.A.); diego.batista@academico.ufpb.br (D.S.B.); figueiredo.uema@gmail.com (F.A.M.M.d.A.F.); fareoli@gmail.com (F.d.O.R.); ferraztm@gmail.com (T.M.F.); 2 Departamento de Engenharia Florestal, Universidade Federal Rural da Amazônia (UFRA), Campus Capitão Poço, Capitão Poço 68650-000, PA, Brazil; sergio.h.s.felipe@gmail.com; 3 Centro de Ciências Agrárias (CCA), Universidade Estadual do Maranhão (UEMA), São Luís 65055-310, MA, Brazil; mnbs2018sh@gmail.com; 4 Programa de Pós-Graduação em Agronomia, Universidade Federal da Paraíba, Areia 58397-000, PB, Brazil; 5 Laboratório de Biologia Celular e Tecidual (LBCT), Centro de Biociências e Biotecnologia (CBB), Universidade Estadual do Norte Fluminense Darcy Ribeiro (UENF), Campos dos Goytacazes 28013-602, RJ, Brazil; laisetmm@gmail.com (L.T.M.M.); claudete@uenf.br (C.S.-C.); 6 Centro de Estudos Superiores de Coelho Neto, Universidade Estadual do Maranhão (UEMA), Coelho Neto 65620-000, MA, Brazil; aldilene29@gmail.com

**Keywords:** anatomical plasticity, in vitro culture, morpho-physiology, polyamines, salinity

## Abstract

*Dizygostemon riparius* is an endemic tropical Brazilian species whose physiological responses to salinity are poorly understood. This study evaluated the effects of NaCl (0, 50, and 100 mM) on in vitro-grown *D. riparius* by integrating growth, anatomical, photosynthetic, and biochemical analyses. Salinity significantly reduced biomass and growth parameters, indicating impaired development. Photosystem II efficiency declined, as evidenced by decreases in the performance index and chlorophyll content, while anatomical changes such as epidermal thickening and reduced vascular bundles reflected structural adjustments under stress. At the biochemical level, salinity altered polyamine metabolism, with reductions in total free polyamines, suggesting potential limitations in defense mechanisms. Hyperhydricity observed under high salinity indicated a non-adaptive response. Overall, *D. riparius* displayed limited tolerance to salt stress, with physiological and biochemical impairments outweighing structural plasticity. This study provides the first data-driven characterization of salinity effects in this species and highlights the value of in vitro culture as a tool to investigate stress responses.

## Simple Summary

*Dizygostemon riparius*, also known as “melosa”, is a subshrub endemic to the Brazilian ‘Cerrado’ with great medicinal and agrochemical potential due to their essential oils, which have biopesticide activity. Due to its recent cataloging, there is a huge gap regarding its cultivation and responses under adverse conditions, such as salinity, including under in vitro conditions—a biotechnological approach widely used in the production of essential oils, which in some cases can be enhanced by salinity. Thus, this study aimed to address how *D. riparius* cultured in vitro responds to salinity stress by evaluating the growth, photosynthesis, and homeostasis of polyamines—a group of plant hormones involved in stress responses. Despite the thickening of the leaf epidermis—a common defense response to salinity—our results indicate that this species is sensitive to salinity, as shown by the impairment of growth and photosynthesis, and decreases in chlorophylls and polyamine contents. These findings pave the way for better understanding the in vitro cultivation of this tropical species, helping to optimize cultivation protocols in saline-prone regions, such as the Tropics, and ultimately contributing to the knowledge about the flora of the Brazilian ‘Cerrado’, a unique and emblematic biome.

## 1. Introduction

*Dizygostemon riparius*, commonly known as “melosa”, is a tropical aromatic subshrub endemic to the northeastern Brazilian Cerrado, which belongs to the Plantaginaceae family [1]. Although recently cataloged, this species has already demonstrated remarkable potential for medicinal and agrochemical applications [2]. Its essential oils and crude extracts exhibit significant larvicidal, acaricidal, and antifungal activities [2,3,4,5], with promising results being obtained in green nanotechnological formulations [2]. Considering the potential of biotechnological techniques for the production of such compounds, it is important to investigate the phenotypic plasticity of *D. riparius* under in vitro conditions, decreasing the knowledge gaps regarding *D. riparus* cultivation, and allowing its domestication and sustainable use.

Salinity, a major abiotic stress that strongly impacts plant growth, can also act as an elicitor in the production of secondary metabolites [6,7]. In tropical regions such as Brazil, salinity is an increasingly critical problem due to irregular rainfall distribution, high evapotranspiration rates, and inadequate irrigation management, which promote the accumulation of soluble salts in agricultural soils [6]. These conditions threaten biodiversity and the productivity of endemic and cultivated species adapted to these ecosystems. This stress primarily disrupts the osmotic balance of cells, reducing the uptake of water and nutrients, and causing ionic toxicity due to the accumulation of Na^+^ and Cl^−^, which compromise membrane integrity, nutrient balance, and DNA stability [6]. Such disturbances stimulate excessive production of reactive oxygen species (ROS), including superoxide anion (O_2_^−^), hydrogen peroxide (H_2_O_2_), and hydroxyl radicals (•OH), ultimately reducing cell viability [8,9,10]. Therefore, understanding the physiological and biochemical responses of endemic tropical species to salinity stress is essential for developing strategies to improve their resilience and conservation under changing environmental conditions.

To counter these effects, plants rely on biochemical defense systems, among which polyamines have emerged as key modulators of developmental and stress-related processes [11]. These small aliphatic amines, such as putrescine (Put), cadaverine (Cad), spermidine (Spd), and spermine (Spm), contribute to membrane stabilization, ion homeostasis, ROS scavenging, and regulation of gene expression [12]. Salinity often reduces Put and Spd, while Cad and Spm show variable responses depending on species and stress intensity [13,14]. Despite their importance, the dynamics of polyamines under salinity remain poorly studied in tropical medicinal plants cultured in vitro.

Disturbances at the cellular and biochemical levels often extend to plant structure and function. Anatomical modifications include epidermal thickening, reduction of palisade and spongy parenchyma, and vascular collapse, which impair hydraulic conductivity and tissue functionality [15]. Physiologically, salinity diminishes chlorophylls and carotenoids, impairs chlorophyll a fluorescence, and compromises photosystem II, resulting in reduced photosynthetic efficiency [16]. These effects culminate in macroscopic symptoms such as chlorosis, necrosis, premature senescence, and growth inhibition, leading to reduced leaf area, impaired stem and root development, and decreased overall biomass production [17,18]. Hormonal imbalances, including increased abscisic acid and reduced auxins and cytokinins, further aggravate these responses by impairing cell division, promoting turgor loss, and restricting development [19].

In pot-grown plants of *D. riparus*, salinity was reported to impair carbon assimilation and dry mass production, with no reductions in photosynthetic pigment content or photosystem II efficiency [20]. Although in vitro culture offers a powerful and controlled platform to investigate plant responses to abiotic stress, no studies to date have characterized the integrated morpho-physiological, anatomical, and biochemical responses of *D. riparius* to salinity under this system. This knowledge gap restricts our understanding of its adaptive capacity and limits its future as a phytotherapeutic and agroindustrial resource derived from tropical vegetation [20].

Here, we hypothesize that salinity impairs growth, tissue structure, and pigment content in *D. riparius* while simultaneously inducing alterations in polyamine metabolism, since these molecules are associated with antioxidant defenses and the regulation of cell division and expansion, which may indicate limited adaptive plasticity under in vitro conditions. Based on this hypothesis, this study aimed to evaluate the effects of salinity on growth parameters, water status, photosystem II integrity, pigment content, anatomical traits, and polyamine metabolism in *Dizygostemon riparius* cultured in vitro.

## 2. Materials and Methods

### 2.1. Plant Cultivation and Experimental Design

The *Dizygostemon riparius* plants used in this experiment originated from the plant collection maintained at the Tissue Culture Laboratory of the State University of Maranhão (LCT/UEMA), MA, Brazil (2°34′00″ S and 44°12′00″ W), since 2022 and subcultured every 90 days. These plants were maintained in vitro under a photomixotrophic system, in transparent 350 mL glass flasks, each containing 60 mL of Murashige and Skoog (MS) medium with vitamins [21] (PhytoTechnology^®^, Lenexa, KS, USA), supplemented with 30 g L^−1^ sucrose (*w*/*v*; Dinâmica^®^ Química Contemporânea Ltda, São Paulo, SP, Brazil) and solidified with 5.5 g L^−1^ agar (Êxodo Científica^®^, São Paulo, SP, Brazil). The cultures were maintained at 25 ± 2 °C, under an irradiance of 85 μmol m^−2^ s^−1^ provided by six white tubular LED lamps (T8, 9 W; Avant, São Paulo, SP, Brazil), with a 16 h photoperiod.

Single-node stem explants approximately 1.5 to 2.0 cm in length were excised from the bank plants and inoculated into 350 mL glass flasks containing 60 mL of MS medium [21] (PhytoTechnology^®^, Lenexa, KS, USA), under the same environmental conditions previously described. The salinity experiment consisted of three treatments with different NaCl concentrations: 0 mM (control), 50 mM, and 100 mM. The flasks were sealed with polypropylene caps containing two 10 mm holes covered with microporous tape membranes, following the methodology described by Saldanha et al. [22]. After inoculation, the flasks were maintained for 45 days in a growth room under an irradiance of 85 μmol m^−2^ s^−1^, with a 16 h photoperiod and a temperature of 25 ± 2 °C. The experimental design was completely randomized, with three salinity treatments and twelve replicates per treatment, and the experimental unit consisted of five explants per flask.

### 2.2. Growth Parameters

After 45 days of in vitro cultivation, shoot length (cm), stem diameter (mm), and the length of the longest root (cm) were measured. Subsequently, leaf area (cm^2^) was determined using ImageJ^®^ software(Version 1.52e). After the measurements, leaves, stems, and roots were sectioned, collected, and placed into pre-labeled paper bags for drying. These plant organs were oven-dried (SolidSteel^®^, Piracicaba, SP, Brazil) at 45 °C until reaching constant weight, and dry mass was then determined for the aerial part (g) and roots (g).

### 2.3. Relative Leaf Water Content (RLWC)

Five leaf disks (3 mm in diameter) were collected from fully expanded leaves in each treatment. The disks were immediately weighed on an analytical balance to determine fresh mass (FM). They were then transferred to 8 mL plastic cups containing distilled water for 24 h to obtain turgid mass (TM). Afterward, the disks were oven-dried at 45 °C for 48 h to determine dry mass (DM). The RLWC was calculated using the formula [23]: RLWC (%) = [(FM − DM)/(TM − DM)] × 100.

### 2.4. Chlorophyll a Fluorescence

Chlorophyll *a* fluorescence measurements were performed on the third pair of fully expanded leaves, counted from the apex toward the base of the stem, at 45 days of in vitro cultivation. A non-modulated fluorometer, model Pocket PEA (Plant Efficiency Analyser, Hansatech^®^, King’s Lynn, UK), was used. Prior to measurement, leaves were dark-adapted for 30 min using leaf clips to ensure that the reaction centers were fully open and heat dissipation was minimized.

The following parameters were evaluated: initial fluorescence (F_0_), maximum fluorescence (Fm), variable fluorescence (Fv), maximum quantum yield of photosystem II (Fv/Fm), energy absorbed per active reaction center (RC/ABS), variable fluorescence relative to initial fluorescence (Fv/F_0_), and performance index (PI).

### 2.5. Extraction and Determination of Photosynthetic Pigment Concentrations

After 45 days of in vitro cultivation, pigments were extracted from three leaf disks, each 3 mm in diameter, collected from the third fully expanded leaf pair counted from the apex to the base of the stem. The disks were placed in test tubes containing 3 mL of dimethyl sulfoxide (DMSO) as the organic extracting solvent and kept in the dark for 48 h, as described by Santos et al. [24].

Absorbance readings were performed using a UV/Vis spectrophotometer (model UV-M51; BEL Engineering, Monza, Italy) at wavelengths of 480, 645, and 665 nm in 10 mm cuvettes. The concentrations of chlorophyll *a*, chlorophyll *b*, chlorophyll total, and carotenoids were calculated using the equations proposed by Wellburn [25]. In addition, the chlorophyll *a*/*b* ratio and the total chlorophyll-to-carotenoid ratio were calculated.

### 2.6. Leaf Thermography

Thermal images of the leaf surface were captured from the third and fourth leaf pairs, counted from the apex to the base of the stem, using a FLIR E8 WIFI thermal camera (FLIR Systems^®^, Wilsonville, OR, USA). The camera was positioned vertically 90 cm from the leaves. Image acquisition was conducted between 08:30 and 09:00 AM (Brasília time, Brazil). The images were processed using FLIR Thermal Studio Suite software, version 2.0.x (Copyright^®^, 2024, Thousand Oaks, CA, USA).

### 2.7. Determination of Polyamine Concentration

Samples of 300 mg of fresh mass were freeze-dried and homogenized with 0.6 mL of 5% (*v*/*v*) perchloric acid (Merck^®^, Darmstadt, Germany), incubated on ice for 1 h, and centrifuged at 16,000× *g* for 20 min at 4 °C. The free polyamines (PAs) were then dansylated. For this, 40 µL of the extract were mixed with 20 µL of 1.7-diaminoheptane (DAH) at 0.05 mM (used as an internal standard), 50 µL of saturated sodium bicarbonate solution (NaHCO_3_), and 100 µL of dansyl chloride (5 mg mL^−1^ in acetone; 1.8 mM) (Merck^®^).

The samples were incubated in the dark at 70 °C for 50 min. Excess dansyl chloride was removed by adding 25 µL of proline solution (100 mg mL^−1^), followed by incubation at room temperature in the dark for 30 min. The dansylated PAs were extracted with 200 µL of toluene, and 175 µL of the organic (non-polar) phase containing the PAs were collected, evaporated under a nitrogen stream, and resuspended in 175 µL of absolute acetonitrile.

Identification and quantification of the PAs were performed using high-performance liquid chromatography (HPLC), with a reverse-phase C18 column (Shimadzu Shim-pack CLC ODS, 5 µm). The mobile phase consisted of 10% acetonitrile in water (pH 3.5, adjusted with 1 N HCl) as solvent A, and absolute acetonitrile as solvent B. Peak areas and retention times of the PAs were compared with commercial standards of putrescine (Put), spermidine (Spd), and spermine (Spm) (Sigma-Aldrich^®^, St. Louis, MO, USA). Polyamines were determined through an estimate of dry mass.

### 2.8. Anatomy and Micromorphometry of Leaf, Stem, and Root

For anatomical characterization, samples of leaf, stem, and root tissues were fixed in a 50% FAA solution (formalin, acetic acid, and ethyl alcohol) following the protocol described by Johansen [26] for 48 h. Subsequently, the samples were dehydrated in a graded ethanol series composed of 30%, 40%, 50%, 60%, and 70% ethanol for one hour each, followed by further dehydration in 80%, 90%, 95%, and 100% ethanol for two hours each at 4 °C. After dehydration, the samples were embedded in Historesin^®^ (Leica Instruments, Heidelberg, Germany), according to the manufacturer’s recommendations. Transverse sections with a thickness of 6 µm were obtained using a rotary microtome (Lupetec^®^ model MRP2015, São Carlos, Brazil). These sections were mounted on glass slides and stained with toluidine blue solution at 0.05% and pH 4.0, as described by O’Brien and McCully [27], for eight minutes.

Micromorphometric analysis was performed on transverse sections of the leaf midrib, stem, and root. From each slide, four anatomical sections were photographed using a light microscope (model B20T; Bioptika, Colombo, PR, Brazil) equipped with a U-photo system and a digital camera (CMOS-5.0; Bioptika, Colombo, PR, Brazil), connected to a computer with Capture V2.1 software. The images were captured at a magnification of 4× and analyzed using the ImageJ^®^ software, with measurements expressed in millimeters. In leaf tissues, the parameters measured included the thickness of the adaxial and abaxial epidermis, the palisade parenchyma, the spongy parenchyma, as well as the diameter of both transverse and longitudinal vascular bundles. In stem tissues, measurements included the thickness of the epidermis, cortical parenchyma, vascular bundles, and medullary parenchyma. In root tissues, the thickness of the epidermis and cortical parenchyma was determined, along with the diameter of the transverse and longitudinal vascular bundles.

### 2.9. Data Analyses

Growth and physiological analyses were performed with 10 replicates per treatment, while polyamine analyses were performed with 3 replicates per treatment. All evaluated variables were tested for homogeneity using Bartlett’s test and for normality with the Shapiro–Wilk test. Subsequently, they were subjected to analysis of variance (ANOVA), and when significant effects were detected, the means were compared using Tukey’s test (*p* ≤ 0.05). The statistical procedures were performed using the Sisvar software (version 5.0) [28].

## 3. Results

### 3.1. Salinity Impairs Growth and Alters Morphoanatomy of Dizygostemon Riparius Cultured In Vitro

**Figure 1:**
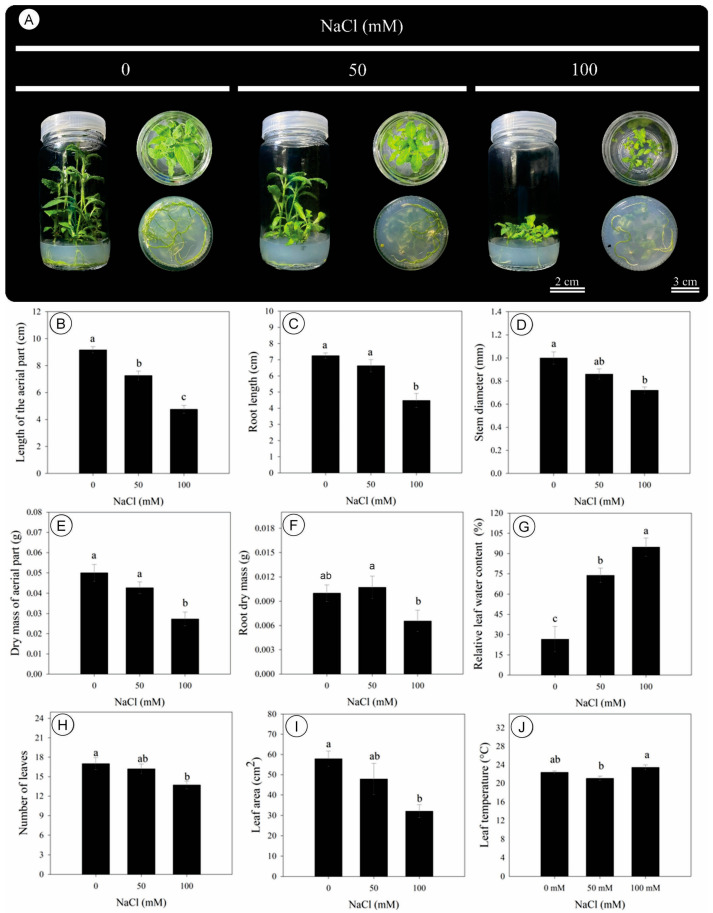
Morphology, growth, and relative leaf water content of *Dizygostemon riparius* under three NaCl concentrations (0, 50, and 100 mM) at 45 days of in vitro cultivation. (**A**) Pictures of representative plants; (**B**) shoot length (cm); (**C**) root length (cm); (**D**) stem diameter (mm); (**E**) shoot dry mass (g); (**F**) root dry mass (g); (**G**) relative leaf water content (%); (**H**) number of leaves; (**I**) leaf area (cm^2^); (**J**) leaf temperature (°C). Different letters above the bars indicate significant differences (*p*  ≤  0.05) according to Tukey’s test. Values are means ± standard error (*n* = 10). Scale for the flasks standing up = 2 cm, and scale for the bottom of the flasks = 3 cm.

Salinity induced pronounced morphological changes in *D. riparius* cultured in vitro after 45 days (Figure 1A). A progressive reduction in the length of the aerial part was observed with increasing NaCl concentration, with statistically significant differences among treatments (Figure 1B). Compared to the control, shoot length was decreased by 21% and 49% under 50 and 100 mM NaCl, respectively (Figure 1B). Root length remained unchanged between 0 and 50 mM but was 40% smaller at 100 mM NaCl (Figure 1C). Stem diameter was also negatively affected, decreasing by 28% under 100 mM NaCl compared to the control (Figure 1D).

The dry mass of the aerial part and roots was decreased by 46% and 40%, respectively, at the highest salinity level (Figure 1E,F). In contrast, relative leaf water content was increased under salinity, reaching its maximum at 100 mM NaCl, corresponding to a 257% increase over the control (Figure 1G). The number of leaves declined by 19% under 100 mM NaCl (Figure 1H), and leaf area was similarly reduced by 45% (Figure 1I). Leaf temperature showed only minor variations across treatments, with a 1.1% increase at 100 mM NaCl, which was not statistically significant (Figure 1J).

**Figure 2:**
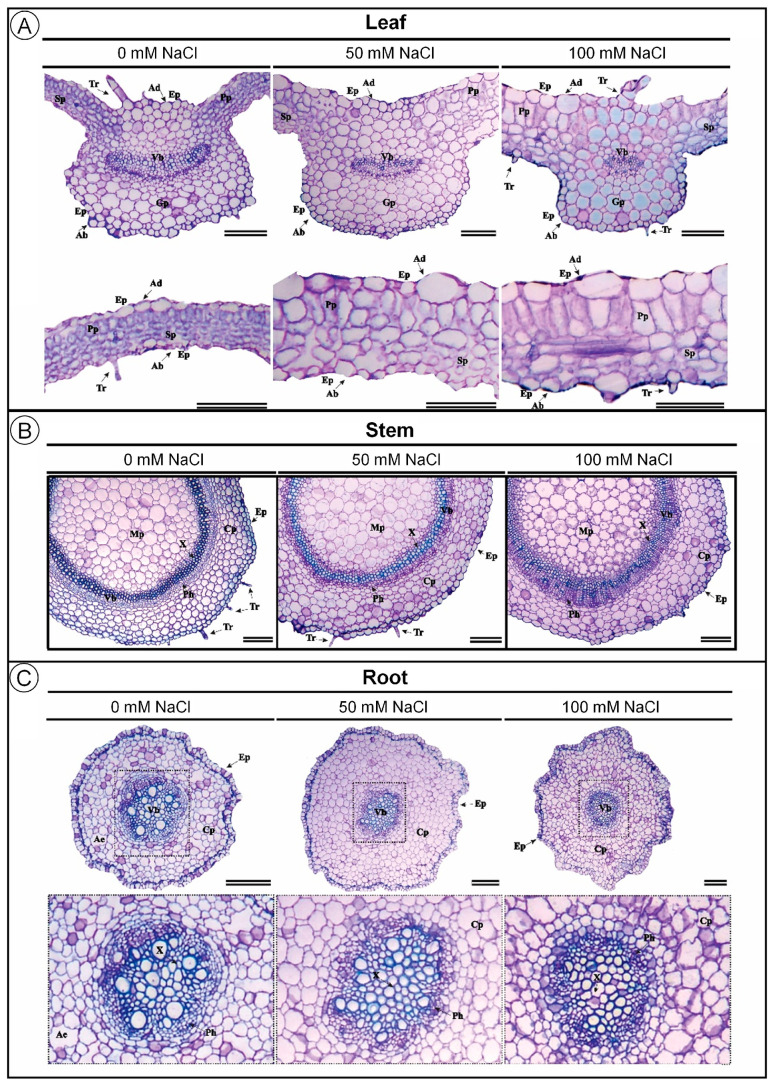
Transverse section micrography of *Dizygostemon riparius* leaves, stems, and roots after 45 days of in vitro cultivation under three NaCl concentrations (0, 50, and 100 mM). (**A**) Transverse sections of the leaf midrib (**up**) and leaf blade (**below**); (**B**) transverse sections of stems; (**C**) transverse sections of roots. Ep = epidermis, Ad = adaxial epidermis, Ab = abaxial epidermis, Vb = vascular bundle, Pp = palisade parenchyma, Sp = spongy parenchyma, Gp = ground parenchyma, X = xylem, Ph = phloem, Cp = cortical parenchyma, Mp = medullary parenchyma, Tr = trichome, and Ae = aerenchyma. Scale bar = 100 µm.

Salinity also induced significant anatomical changes in leaves and roots of *D. riparius* (Figure 2A,C). The adaxial epidermis thickness increased with rising NaCl concentrations, with increments of approximately 46% at 50 mM and 75% at 100 mM compared to the control (Table 1). In contrast, the abaxial epidermis remained statistically unchanged across treatments. Palisade parenchyma thickness, in turn, was slightly increased under salinity, with values rising by 3% and 6% at 50 and 100 mM NaCl, respectively (Table 1). Spongy parenchyma was also increased by about 4% under both salinity levels, compared to the control. In the vascular system, transverse bundle thickness was progressively decreased with increasing salinity, with a 13% reduction at 100 mM NaCl compared to the control (Table 1). However, longitudinal vascular bundle thickness was not significantly affected.

Unlike the leaf, stem anatomy of *D. riparius* remained structurally stable under salinity (Figure 2B; Table 1). No significant differences were observed in epidermis thickness, cortical parenchyma, vascular bundle thickness, or medullary parenchyma, indicating high anatomical resilience of stem tissues even under moderate to high salinity conditions.

In contrast, salinity significantly altered root anatomy, particularly the vascular system (Figure 2C; Table 1). Transverse vascular bundle thickness was progressively decreased with increasing NaCl, showing a 48.57% reduction at 100 mM compared to the control. This reduction was statistically significant, indicating root vascular sensitivity to elevated salinity. Meanwhile, epidermis thickness, cortical parenchyma, and longitudinal vascular bundle thickness remained statistically unchanged.

### 3.2. Salinity Decreases Photosynthetic Pigment Content and Chlorophyll a Fluorescence in Dizygostemon Riparius

**Figure 3:**
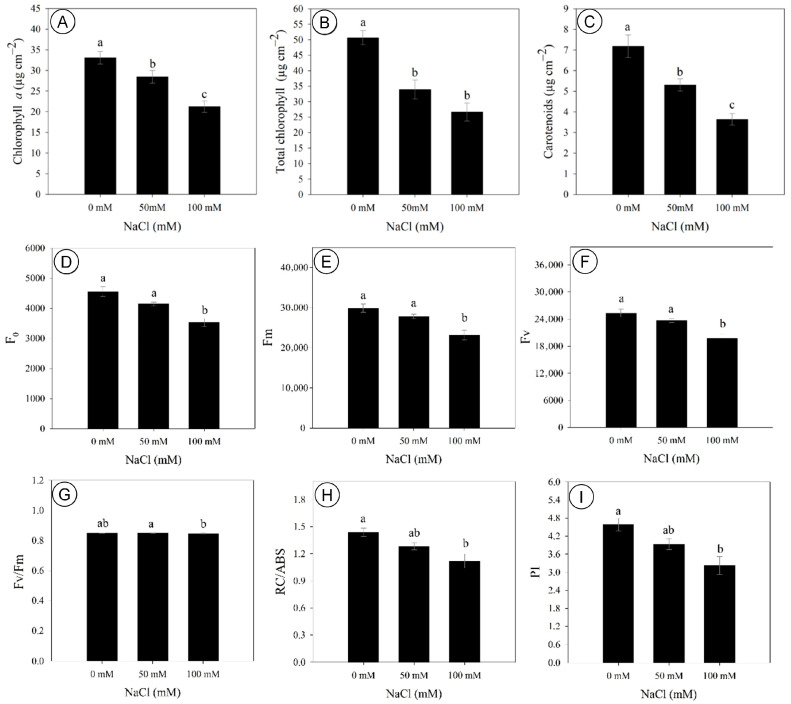
Photosynthetic pigments and chlorophyll *a* fluorescence parameters of *Dizygostemon riparius* at 45 days of in vitro cultivation under three NaCl concentrations (0, 50, and 100 mM). (**A**) Chlorophyll *a* (µg cm^−2^); (**B**) total chlorophyll (µg cm^−2^); (**C**) carotenoids (µg cm^−2^); (**D**) initial fluorescence (F_0_); (**E**) maximum fluorescence (F_m_); (**F**) variable fluorescence (F_v_); (**G**) maximum quantum yield of photosystem II (F_v_/F_m_); (**H**) absorbed energy per active reaction center (RC/ABS); and (**I**) performance index (PI). Different letters above the bars indicate significant differences (*p*  ≤  0.05) according to Tukey’s test. Values are means ± standard error (*n* = 10).

The contents of photosynthetic pigments in *D. riparius* were significantly affected by salinity, with chlorophyll *a* content being decreased by 14% and 36% under 50 and 100 mM NaCl, respectively, compared to the control (Figure 3A). Similarly, the content of total chlorophylls had a marked reduction of 36% at 50 mM and 42% at 100 mM NaCl, compared to the control (Figure 3B). Carotenoid content was also negatively affected, with decreases of 26% at 50 mM and 50% at 100 mM, compared to control (Figure 3C). On the other hand, chlorophyll *b* content, chlorophyll *a/b* ratio, and total chlorophyll-to-carotenoid ratio were not affected by salinity.

Chlorophyll *a* fluorescence was significatively impaired by 100 mM NaCl, with the initial fluorescence (F_0_), maximum fluorescence (F_m_), and variable fluorescence (F_v_) being reduced by 29%, 23%, and 22%, respectively, compared to the control (Figure 3D–F). The F_v_/F_m_ ratio of salt-stressed plants did not differ from the control, although plants under 50 mM exhibited slightly higher values compared to 100 mM NaCl (Figure 3G). In turn, the number of active reaction centers per absorbed energy unit (RC/ABS) and the performance index (PI) were decreased by 22% and 30% at 100 mM, respectively (Figure 3H,I). The F_v_/F_0_ ratio was not affected by salinity levels.

### 3.3. Salinity Modulates Endogenous Polyamine Levels in Dizygostemon Riparius Cultured In Vitro

**Figure 4:**
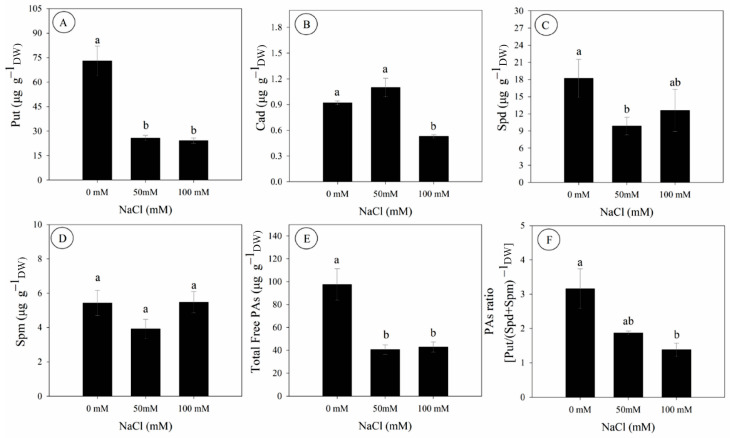
Endogenous polyamine content (determined by dry mass) in *Dizygostemon riparius* at 45 days of in vitro cultivation under three NaCl concentrations (0, 50, and 100 mM). (**A**) Putrescine (µg g^−1^); (**B**) Cadaverine (µg g^−1^); (**C**) Spermidine (µg g^−1^); (**D**) Spermine (µg g^−1^); (**E**) Total free polyamines (µg g^−1^); and (**F**) Polyamine ratio [Put/(Spd + Spm)]. Different letters above the bars indicate significant differences (*p*  ≤  0.05) according to Tukey’s test. Values are means ± standard error (*n* = 3).

Salinity significantly affected the contents of free endogenous polyamines in *D. riparius* cultured in vitro (Figure 4). Compared to the control, the contents of putrescine (Put) and cadaverine (Cad) were decreased by 67% and 42% at 100 mM NaCl, respectively, while spermidine (Spd) was decreased by 46% at 50 mM NaCl (Figure 4A–C). In contrast, spermine (Spm) contents remained unchanged across treatments (Figure 4D).

Total free polyamines were significantly lower under salinity, with reductions of 59% and 57% under 50 and 100 mM NaCl, respectively (Figure 4E). Similarly, the PAs ratio [Put/(Spd + Spm)], commonly used as a metabolic stress indicator, was decreased by 56% at 100 mM NaCl compared to the control (Figure 4F).

## 4. Discussion

Salinity compromised the growth of both aerial and root organs, mainly under the highest NaCl concentration (100 mM). Shoot length, leaf number, leaf area, stem diameter, and dry biomass were all decreased under high NaCl concentrations. This growth inhibition is likely related to salt-induced osmotic stress and ionic toxicity, as they are known to inhibit cell division and elongation, decrease meristematic activity, and impair the deposition of cellulose and lignin [6]. The impairment in stem and leaf development also disrupts sap transport and compromises the physiological performance [29,30]. Root growth also declined under 100 mM NaCl, likely due to ionic toxicity and oxidative stress in the root zone [6]. Moreover, root growth inhibition might be related to the salinity-induced reduction in water potential of the medium, which impairs nutrient and water uptake and leads to nutritional imbalances [31,32,33].

Anatomical responses highlighted both compensatory mechanisms and structural damages. The adaxial epidermis was thickened in response to salinity, which may be a protective barrier against osmotic stress by reducing transpiration and restricting ion entry [34]. Palisade and spongy parenchyma were also thickened, suggesting an attempt to maintain photosynthetic capacity and partially compensate for pigment loss and reduced PSII efficiency [35,36]. In contrast, the transverse vascular bundles in both leaves and roots became thinner under high salinity, suggesting impaired transport of water and nutrients. This reduction likely resulted from cell retraction, vascular collapse, or stress-induced deposition of lignin and suberin [37,38].

Relative water content was markedly increased under 100 mM NaCl, which could initially suggest osmotic adjustments. In general, plants maintain cellular turgor in saline environments by accumulating compatible solutes such as sugars, amino acids, and inorganic ions, which balance water potential and protect cellular structures [39,40,41]. In this study, however, the exceptionally high RWC values revealed hyperhydricity induced by salinity. This physiological disorder, commonly observed in in vitro conditions, involves excessive water accumulation in tissues, resulting in a translucent appearance, aqueous consistency, and structural abnormalities [42,43]. A high NaCl concentration is likely to trigger osmotic imbalances and altered membrane permeability, thereby favoring this phenomenon. Thus, although the data show water retention, this scenario represents a maladaptive stress response rather than a functional adaptation.

Salinity also altered the content and proportion of photosynthetic pigments. Chlorophyll *a* and total chlorophyll declined, suggesting pigment degradation probably through ROS that damage thylakoid membranes and pigment-protein complexes [44,45], and salinity-induced inhibition of chlorophyll biosynthesis by targeting key enzymes such as ALA synthase (5-aminolevulinic acid) [46]. As carotenoids dissipate excess light energy and scavenge ROS such as singlet oxygen and peroxyl radicals, their reduction likely increases cellular vulnerability to photoinhibition and lipid peroxidation [47,48]. Thus, low carotenoid contents under salinity indicate impaired non-enzymatic antioxidant defenses. The unchanged chlorophyll a/b and chlorophyll/carotenoid ratios suggest proportional pigment degradation, indicating that both photosystems and their associated light-harvesting complexes were similarly affected. These results differ from those of Albuquerque et al. [20], who reported unexpectedly high pigment levels in pot-grown *D. riparius* under salinity. Differences in stress intensity or exposure duration may explain this discrepancy, since both factors directly influence pigment dynamics.

Chlorophyll *a* fluorescence, a sensitive tool for evaluating the integrity and efficiency of photosystem II (PSII), was also strongly affected by salinity. The decreases in maximum fluorescence (F_m_) under 100 mM NaCl indicate damages to reaction centers and impaired energy coupling between antenna pigments and PSII [49]. Initial fluorescence (F_0_) was also decreased, indicating irreversible inactivation of PSII centers [50], while the decline in active reaction centers per absorption (RC/ABS) corresponds to decreases in functional PSII density [51]. This likely resulted from inhibited protein synthesis, particularly of D1, a primary target of degradation under stress, as well as increased oxidative damage in thylakoid membranes [45,52]. The drop in performance index (PI) further confirmed reduced PSII vitality and potential collapse of the electron transport chain [53].

Salinity significantly reduced the endogenous levels of putrescine and spermidine, while cadaverine only declined at higher concentrations, resulting in a decrease in the total content of free polyamines and indicating a disruption in nitrogen metabolism. Plants synthesize putrescine from L-ornithine via ODC or from L-arginine through arginine decarboxylase (ADC), followed by agmatine, with both pathways driving the initial stages of PA biosynthesis [54,55]. Thus, the sharp decline in putrescine may reflect the inhibition of ornithine decarboxylase (ODC) or precursor molecules such as L-arginine. The reductions in cadaverine and spermidine may also reflect a metabolic bottleneck or enhanced degradation of polyamines as a stress response [56].

The decreased Put/(Spd + Spm) ratio indicates that *D. riparius* experienced an imbalance in polyamine dynamics under higher salinity levels, potentially due to metabolic diversion of putrescine toward conjugation or oxidation. The pronounced reduction in total free polyamines at both 50 and 100 mM NaCl may have contributed to physiological constraints such as impaired cell elongation, disrupted ion homeostasis, and altered water relations, which are likely related to the hyperhydricity observed under high salinity [57,58,59,60]. While these interpretations are based on species-specific observations, we did not measure oxidative stress markers; thus, the proposed interplay between polyamine reduction and hyperhydricity remains hypothetical and should be further explored in future studies.

In summary, salinity reduced growth, biomass accumulation, and anatomical integrity of *D. riparius*, impairing both shoot and root development. Photosynthetic efficiency declined due to pigment degradation and reduced PSII activity, particularly through losses of chlorophylls and carotenoids. Salinity also disrupted polyamine metabolism, with marked reductions in putrescine, spermidine, and total free PAs, and a decline in cadaverine under higher stress, all of which indicate nitrogen metabolism imbalance and low adaptive capacity. Anatomical modifications, such as epidermal thickening and expanded parenchyma, indicate partial compensatory responses that may support limited homeostasis under moderate salinity conditions.

Importantly, this study provides the first evidence of salinity-induced alterations in polyamine metabolism and anatomical plasticity in *D. riparius*, an endemic medicinal species of the Brazilian semiarid region. Unlike widely studied model or crop species, *D. riparius* thrives under naturally fluctuating osmotic conditions, making it a valuable model for understanding how polyamine-mediated mechanisms contribute to stress tolerance in plants adapted to seasonally saline tropical ecosystems. By characterizing these responses, our findings broaden the physiological basis for exploring the ecological resilience and potential biotechnological uses of native tropical species under increasing salinity pressures.

## 5. Conclusions

Our findings demonstrated that *Dizygostemon riparius*, when cultivated in vitro, exhibits limited adaptive responses under increasing salinity. Salinity impaired shoot and root development, reduced biomass accumulation, and altered vascular structure. Physiological evaluations revealed decreased photosynthetic performance, pigment loss, and symptoms of hyperhydricity. Biochemically, the marked decline in endogenous polyamines, particularly putrescine, cadaverine, and spermidine, indicates disruption of polyamine metabolism. Although structural adjustments such as adaxial epidermal thickening and mesophyll expansion occurred, these changes were not sufficient to sustain functional balance under elevated salt concentrations.

These findings enhance our understanding of salinity responses in a poorly studied medicinal tropical species, highlighting its potential use in in vitro screening, clonal propagation, and conservation under controlled conditions. Moreover, the generated data may guide future efforts to optimize cultivation protocols in saline-prone regions, such as the Tropics, and support the selection of tolerant genotypes for ex situ conservation and biotechnological applications.

## Figures and Tables

**Table 1:** Micromorphometric parameters of *Dizygostemon riparius* leaves, stems, and roots after 45 days of in vitro cultivation under three NaCl concentrations (0, 50, and 100 mM).

Organ	Variables (mm)	0 mM NaCl	50 mM NaCl	100 mM NaCl
Leaf	Adaxial epidermis	0.024 ± 0.002 c	0.035 ± 0.003 b	0.042 ± 0.006 a
	Abaxial epidermis	0.019 ± 0.005 a	0.024 ± 0.005 a	0.025 ± 0.004 a
	Palisade parenchyma	0.032 ± 0.009 c	0.053 ± 0.003 b	0.081 ± 0.017 a
	Spongy parenchyma	0.044 ± 0.004 b	0.088 ± 0.008 a	0.087 ± 0.007 a
	Transv. vascular bundle	0.303 ± 0.052 a	0.218 ± 0.035 ab	0.195 ± 0.014 b
	Long. vascular bundle	0.102 ± 0.021 a	0.076 ± 0.018 a	0.065 ± 0.005 a
Stem	Epidermis	0.027 ± 0.009 a	0.027 ± 0.004 a	0.027 ± 0.003 a
	Cortical parenchyma	0.183 ± 0.011 a	0.186 ± 0.042 a	0.194 ± 0.011 a
	Vascular bundle	0.087 ± 0.012 a	0.074 ± 0.022 a	0.082 ± 0.008 a
	Medullary parenchyma	0.336 ± 0.033 a	0.314 ± 0.019 a	0.308 ± 0.016 a
Root	Epidermis	0.038 ± 0.023 a	0.037 ± 0.008 a	0.032 ± 0.009 a
	Cortical parenchyma	0.229 ± 0.083 a	0.293 ± 0.010 a	0.246 ± 0.022 a
	Transv. vascular bundle	0.355 ± 0.063 a	0.276 ± 0.012 ab	0.182 ± 0.034 b
	Long. vascular bundle	0.333 ± 0.055 a	0.314 ± 0.053 a	0.231 ± 0.031 a

Values are means ± standard error of 4 individuals. Different letters indicate significant differences (*p*  ≤  0.05) among NaCl concentrations (lines) according to Tukey’s test. Transv = Tranverse; Long = Longitudinal.

## Data Availability

Data will be made available on reasonable request to the corresponding authors.

## Abbreviations

The following abbreviations are used in this manuscript:

NaCl	Sodium chloride

## References

[1] Scatigna A.V., Brandão C.M., Dalla Colletta G., Teles R.D.M., Cavalcante K.S.B., Souza V.C., Simões A.O. (2019). Dizygostemon riparius (Plantaginaceae, Gratioleae), a new species from Maranhão, northeastern Brazil. Willdenowia.

[2] Brandão C.M., Cavalcante K.S.B., Teles R.M., Marques G.E.C., Monteiro O.S., Andrade E.H.A., Maia J.G.S. (2020). Composition and larvicidal activity of the oil of *Dizygostemon riparius* (Plantaginaceae), a new aromatic species occurring in Maranhão, Brazil. Chem. Biodivers..

[3] Martins S.M.A., Cavalcante K.S.B., Teles R.M., Brandão C.M., Godinho A.S., Silva L.K., Rocha C.Q. (2023). Chemical profiling of *Dizygostemon riparius* (Plantaginaceae) plant extracts and its application against larvae of *Aedes aegypti* L. (Diptera: Culicidae). Acta Trop..

[4] Corrêa L.A.D., Rodrigues A.A.C., Dias L.R.C., Silva E.K.C.E., Monteiro O.D.S., Oliveira L.D.J.M.G.D. (2023). Antifungal potential of essential oils from *Pectis brevipedunculata* and *Dizygostemon riparius* in anthracnose control in mango. Rev. Bras. Frutic..

[5] Ferreira M.C., Nojosa E.C.N., Marques I.S., Brandão C.M., Santos D.R., Carvalho Marques G.E., Barbosa D.R.E.S. (2025). Bioactivity of essential oils of *Dizygostemon riparius* (Plantaginaceae) on *Tetranychus neocaledonicus* (Acari: Tetranychidae). Phytoparasitica.

[6] Atta K., Mondal S., Gorai S., Singh A.P., Kumari A., Ghosh T., Jespersen D. (2023). Impacts of salinity stress on crop plants: Improving salt tolerance through genetic and molecular dissection. Front. Plant Sci..

[7] Reshi Z.A., Ahmad W., Lukatkin A.S., Javed S.B. (2023). From nature to lab: A review of secondary metabolite biosynthetic pathways, environmental influences, and in vitro approaches. Metabolites.

[8] Tsai Y.C., Chen K.C., Cheng T.S., Lee C., Lin S.H., Tung C.W. (2019). Chlorophyll fluorescence analysis in diverse rice varieties reveals the positive correlation between the seedlings salt tolerance and photosynthetic efficiency. BMC Plant Biol..

[9] Sahu M., Maurya S., Jha Z. (2023). In vitro selection for drought and salt stress tolerance in rice: An overview. Plant Physiol. Rep..

[10] Habibi N., Aryan S., Sediqui N., Terada N., Sanada A., Kamata A., Koshio K. (2025). Enhancing salt tolerance in tomato plants through PEG6000 seed priming: Inducing antioxidant activity and mitigating oxidative stress. Plants.

[11] Pallavi S., Chauhan S., Karuna I.S., Malik A. (2025). Polyamines and plant growth regulators: The dynamic duo alleviating salinity stress in plants. J. Integr. Biol. Sci..

[12] Blázquez M.A. (2024). Polyamines: Their role in plant development and stress. Annu. Rev. Plant Biol..

[13] Todorova D., Katerova Z., Sergiev I., Alexieva V. (2012). Role of polyamines in alleviating salt stress. Ecophysiology and Responses of Plants Under Salt Stress.

[14] Lechowska K., Wojtyla Ł., Quinet M., Kubala S., Lutts S., Garnczarska M. (2021). Endogenous polyamines and ethylene biosynthesis in relation to germination of osmoprimed *Brassica napus* seeds under salt stress. Int. J. Mol. Sci..

[15] Jiang L., Wu D., Li W., Liu Y., Li E., Li X., He X. (2024). Variations in physiological and biochemical characteristics of *Kalidium foliatum* leaves and roots in two saline habitats in desert region. Forests.

[16] Waheed A., Zhuo L., Wang M., Hailiang X., Tong Z., Wang C., Aili A. (2024). Integrative mechanisms of plant salt tolerance: Biological pathways, phytohormonal regulation, and technological innovations. Plant Stress.

[17] Molnar S., Clapa D., Hârța M., Andrecan F.A., Bunea C.I. (2024). Investigation of salinity tolerance to different cultivars of highbush blueberry (*Vaccinium corymbosum* L.) grown in vitro. Not. Bot. Horti Agrobot. Cluj-Napoca.

[18] Diallo B., Diatta S., Founoune-Mboup H., Fall A.F., Sane D. (2024). In vitro morpho-physiological characterization of cassava accessions according to salinity tolerance. Biotechnol. Agron. Soc. Environ..

[19] Shi B., Li K., Xu R., Zhang F., Yu Z., Ding Z., Tian H. (2025). Methionine-mediated compensation between plant growth and salt tolerance. Plant Physiol..

[20] Albuquerque I.C., Silva-Moraes V.K.D.O., Alves G.L., Pinheiro J.F., Henschel J.M., Lima A.D.S., Felipe S.H.S. (2024). The role of salicylic acid in salinity stress mitigation in *Dizygostemon riparius*: A medicinal species native to South America. Plants.

[21] Murashige T., Skoog F. (1962). A revised medium for rapid growth and bio assays with tobacco tissue cultures. Physiol. Plant..

[22] Saldanha C.W., Otoni C.G., de Azevedo J.L.F., Dias L.L.C., do Rêgo M.M., Otoni W.C. (2012). A low-cost alternative membrane system that promotes growth in nodal cultures of Brazilian ginseng [*Pfaffia glomerata* (Spreng.) Pedersen]. Plant Cell Tissue Organ Cult..

[23] Barrs H.D., Weatherley P.E. (1962). A re-examination of the relative turgor technique for estimating water deficits in leaves. Aust. J. Biol. Sci..

[24] Santos R.P., da Cruz A.C.F., Iarema L., Kuki K.N., Otoni W.C. (2008). Protocolo para extração de pigmentos foliares em porta-enxertos de videira micropropagados. Rev. Ceres.

[25] Wellburn A.R. (1994). The spectral determination of chlorophylls a and b, as well as total carotenoids, using various solvents with spectrophotometers of different resolution. J. Plant Physiol..

[26] Johansen D.A. (1940). Plant Microtechnique.

[27] O’Brien T.P., McCully M.E. (1981). The Study of Plant Structure: Principles and Selected Methods.

[28] Ferreira D.F. (2011). Sisvar: Um sistema computacional de análise estatística. Cienc. Agrotec..

[29] Munns R., Tester M. (2008). Mechanisms of salinity tolerance. Annu. Rev. Plant Biol..

[30] Fu H., Yang Y. (2023). How plants tolerate salt stress. Curr. Issues Mol. Biol..

[31] Hassan N.E. (2024). Salinity stress in plants: Growth, photosynthesis and adaptation review. GSC Adv. Res. Rev..

[32] Rai G.K., Mishra S., Chouhan R., Mushtaq M., Chowdhary A.A., Rai P.K., Gandhi S.G. (2023). Plant salinity stress, sensing, and its mitigation through WRKY. Front. Plant Sci..

[33] Hostetler A.N., Morais de Sousa Tinoco S., Sparks E.E. (2024). Root responses to abiotic stress: A comparative look at root system architecture in maize and sorghum. J. Exp. Bot..

[34] Shams M., Khadivi A. (2023). Mechanisms of salinity tolerance and their possible application in the breeding of vegetables. BMC Plant Biol..

[35] Li S., Lu S., Wang J., Chen Z., Zhang Y., Duan J., Guo J. (2023). Responses of physiological, morphological and anatomical traits to abiotic stress in woody plants. Forests.

[36] Tang J., Ji X., Li A., Zheng X., Zhang Y., Zhang J. (2024). Effect of persistent salt stress on the physiology and anatomy of hybrid walnut (*Juglans major* × *Juglans regia*) seedlings. Plants.

[37] Keshavarzi M., Esna-Ashari M. (2022). Anatomical changes in root and aerial organs of grapevine (*Vitis vinifera* Cv. Yaghooti) affected by salinity. Plant Prod..

[38] Zhang R., Wang Y., Wang X., Jiao S., Lu Y., Du Y., Qin S. (2025). Differential responses of microstructure, antioxidant defense, and plant hormone signaling regulation in potato (*Solanum tuberosum* L.) under drought, alkaline salt, and combined stresses. Sci. Hortic..

[39] Silva E.N.D., Silveira J.A.G., Rodrigues C.R.F., Lima C.S.D., Viégas R.A. (2009). Contribution of organic and inorganic solutes to osmotic adjustment of physic nut under salinity. Pesqui. Agropecu. Bras..

[40] Turner N.C. (2018). Turgor maintenance by osmotic adjustment: 40 years of progress. J. Exp. Bot..

[41] Singh P., Choudhary K.K., Chaudhary N., Gupta S., Sahu M., Tejaswini B., Sarkar S. (2022). Salt stress resilience in plants mediated through osmolyte accumulation and its crosstalk mechanism with phytohormones. Front. Plant Sci..

[42] Polivanova O.B., Bedarev V.A. (2022). Hyperhydricity in plant tissue culture. Plants.

[43] Bethge H., Mohammadi Nakhjiri Z., Rath T., Winkelmann T. (2023). Towards automated detection of hyperhydricity in plant in vitro culture. Plant Cell Tissue Organ Cult..

[44] Lu W., Wei G., Zhou B., Liu J., Zhang S., Guo J. (2022). A comparative analysis of photosynthetic function and reactive oxygen species metabolism responses in two hibiscus cultivars under saline conditions. Plant Physiol. Biochem..

[45] Stefanov M.A., Rashkov G.D., Borisova P.B., Apostolova E.L. (2024). Changes in photosystem II complex and physiological activities in pea and maize plants in response to salt stress. Plants.

[46] Wu Y., Jin X., Liao W., Hu L., Dawuda M.M., Zhao X., Yu J. (2018). 5-Aminolevulinic acid (ALA) alleviated salinity stress in cucumber seedlings by enhancing chlorophyll synthesis pathway. Front. Plant Sci..

[47] Surówka E., Latowski D., Dziurka M., Rys M., Maksymowicz A., Żur I., Miszalski Z. (2021). ROS-scavengers, osmoprotectants and violaxanthin de-epoxidation in salt-stressed *Arabidopsis thaliana* with different tocopherol composition. Int. J. Mol. Sci..

[48] Homayouni H., Razi H., Izadi M., Alemzadeh A., Kazemeini S.A., Niazi A., Vicente O. (2024). Temporal changes in biochemical responses to salt stress in three *Salicornia* species. Plants.

[49] Makhtoum S., Sabouri H., Gholizadeh A., Ahangar L., Katouzi M., Mastinu A. (2023). Genomics and physiology of chlorophyll fluorescence parameters in *Hordeum vulgare* L. under drought and salt stresses. Plants.

[50] Guidi L., Lo Piccolo E., Landi M. (2019). Chlorophyll fluorescence, photoinhibition and abiotic stress: Does it make any difference the fact to be a C3 or C4 species?. Front. Plant Sci..

[51] Yan K., Mei H., Dong X., Zhou S., Cui J., Sun Y. (2022). Dissecting photosynthetic electron transport and photosystems performance in Jerusalem artichoke (*Helianthus tuberosus* L.) under salt stress. Front. Plant Sci..

[52] Lin S., Song X.F., Mao H.T., Li S.Q., Gan J.Y., Yuan M., Chen Y.E. (2022). Exogenous melatonin improved photosynthetic efficiency of photosystem II by reversible phosphorylation of thylakoid proteins in wheat under osmotic stress. Front. Plant Sci..

[53] Hammami Z., Tounsi-Hammami S., Nhamo N., Rezgui S., Trifa Y. (2024). The efficiency of chlorophyll fluorescence as a selection criterion for salinity and climate aridity tolerance in barley genotypes. Front. Plant Sci..

[54] Kusano T., Berberich T., Tateda C., Takahashi Y. (2008). Polyamines: Essential factors for growth and survival. Planta.

[55] Napieraj N., Reda M., Janicka M. (2020). The role of NO in plant response to salt stress: Interactions with polyamines. Funct. Plant Biol..

[56] González-Hernández A.I., Scalschi L., Vicedo B., Marcos-Barbero E.L., Morcuende R., Camañes G. (2022). Putrescine: A key metabolite involved in plant development, tolerance and resistance responses to stress. Int. J. Mol. Sci..

[57] Upadhyay R.K., Fatima T., Handa A.K., Mattoo A.K. (2021). Differential association of free, conjugated, and bound forms of polyamines and transcript abundance of their biosynthetic and catabolic genes during drought/salinity stress in tomato (*Solanum lycopersicum* L.) leaves. Front. Plant Sci..

[58] Samanta I., Roy P.C., Das E., Mishra S., Chowdhary G. (2023). Plant peroxisomal polyamine oxidase: A ubiquitous enzyme involved in abiotic stress tolerance. Plants.

[59] El-Beltagi H.S., El-Yazied A.A., El-Gawad H.G.A., Kandeel M., Shalaby T.A., Mansour A.T., Ibrahim M.F. (2023). Synergistic impact of melatonin and putrescine interaction in mitigating salinity stress in snap bean seedlings: Reduction of oxidative damage and inhibition of polyamine catabolism. Horticulturae.

[60] Borromeo I., Domenici F., Del Gallo M., Forni C. (2023). Role of polyamines in the response to salt stress of tomato. Plants.

